# Antibiotics and Antibiotic Resistance Genes in the Environment: Dissemination, Ecological Risks, and Remediation Approaches

**DOI:** 10.3390/microorganisms13081763

**Published:** 2025-07-29

**Authors:** Zhaomeng Wu, Xiaohou Shao, Qilin Wang

**Affiliations:** 1 College of Agricultural Science and Engineering, Hohai University, Nanjing 211100, China; wuzhaomeng2833@163.com (Z.W.); wql231024@163.com (Q.W.); 2 Key Laboratory of Environment Remediation and Ecological Health, Ministry of Education, Zhejiang University, Hangzhou 310058, China

**Keywords:** novel environmental pollutants, environmental contamination, horizontal gene transfer, adsorption, advanced oxidation processes, biological degradation

## Abstract

Global antibiotic use saturates ecosystems with selective pressure, driving mobile genetic element (MGE)-mediated antibiotic resistance gene (ARG) dissemination that destabilizes ecological integrity and breaches public health defenses. This review synthesizes the sources, environmental distribution, and ecological risks of antibiotics and ARGs, emphasizing the mechanisms of horizontal gene transfer (HGT) driven by MGEs such as plasmids, transposons, and integrons. We further conduct a comparative critical analysis of the effectiveness and limitations of antibiotics and ARGs remediation strategies for adsorption (biochar, activated carbon, carbon nanotubes), chemical degradation (advanced oxidation processes, Fenton-based systems), and biological treatment (microbial degradation, constructed wetlands). To effectively curb the spread of antimicrobial resistance and safeguard the sustainability of ecosystems, we propose an integrated “One Health” framework encompassing enhanced global surveillance (antibiotic residues and ARGs dissemination) as well as public education.

## 1. Introduction

Antibiotics play a critical role in disease prevention; currently, more than 600 antibiotics are used in clinical practice to treat diseases and protect public health. It is projected that the usage of antibiotics will reach 75.1 billion limited daily doses (DDDs) by 2030, an increase of 52.3% compared to the level in 2023 [1]. Based on their chemical structure and action mechanism, antibiotics can be classified into *β*-lactams, tetracyclines (TCs), quinolones (QNs), sulfonamides (SAs), macrolides (MLs), and aminoglycosides (AGs) [2]. Antibiotics have been extensively utilized in aquaculture and animal husbandry [3,4]. However, most antibiotics are not completely absorbed by animals and are excreted into the environment in the form of original drugs or active metabolites through urine or feces, leading to environmental pollution [5,6,7,8,9]. Given their characteristics of refractory degradation, high bio-accumulation and unknown toxicity, antibiotics have emerged as new pollutants in the global water and soil environment [10]. A variety of antibiotics have been frequently detected in surface water, groundwater, soil, and sediments, making the monitoring of antibiotic residues critically important across various fields.

The annual global consumption of antibiotics ranges from 100,000 to 200,000 tons, with China alone exceeding 25,000 tons [11]. Epidemiological studies have demonstrated that the excessive use of antibiotics can exacerbate the emergence of bacterial resistance, leading to a significant public health crisis [11,12]. Without effective interventions, antimicrobial resistance (AMR)-related deaths could exceed 10 million by 2050 [13,14]. To address the AMR crisis, the World Health Organization (WHO) has established a classification system for antibacterial drugs, aiming to optimize the use strategies to reduce the risk of AMR. Meanwhile, developing countries are also facing the problem of serious environmental pollution caused by antibiotics [12].

The misuse of antibiotics has significantly accelerated the horizontal transmission of antibiotic resistance genes (ARGs) [15]. Numerous studies have confirmed that ARGs can be transferred among bacterial species through mobile genetic elements (MGEs), such as plasmids [16], transposons [17], and integrons [18]. This transfer particularly relies on the bacterial type IV secretion system (T4SS) [19]. As a conserved, multifunctional transmembrane channel found in both Gram-negative and Gram-positive bacteria, T4SS mediates the transfer of ARG-bearing plasmids via conjugation, enabling the genetic materials associated with drug resistance to enter recipient cells and leading to the emergence of multidrug-resistant (MDR) bacteria [20,21]. Through the transformation of T4SS, bacteria can uptake DNA or ARGs from the external environment, thereby promoting the spread of ARGs [22]. These results highlight the crucial role of T4SS in exacerbating the global AMR crisis, and the crisis will continue to intensify and pose a significant threat to human health if not effectively addressed to control or eliminate ARGs.

This paper examines the current pollution situations, the transmission mechanisms, and the treatment strategies for antibiotics and ARGs; summarizes the latest developments in adsorption, advanced oxidation processes (AOPs), and microbial degradation technologies; and discusses the limitations of existing mitigation technologies and future prospects. The aim of this review is to provide theoretical guidance and a scientific foundation for eliminating antibiotic pollution and preventing the spread of ARGs in the environment.

## 2. Overview of Antibiotic Contamination in the Environment

### 2.1. Current Status of Using Antibiotics

There has been a significant escalation in global antibiotic consumption since 2000. Between 2000 and 2015, worldwide utilization of antibiotics increased by 65%. This surge was primarily attributed to low- and middle-income countries, where per capita consumption rose by 76%, in stark contrast to a 4% decrease observed in high-income countries during the same period [23]. From 2016 to 2023, global antibiotic usage further increased by 20.9%, and it would rise by 52.3% in 2030 without the implementation of intervention strategies [1]. It is noteworthy that the inappropriate use of antibiotics during the COVID-19 pandemic has exacerbated this trend [12,24].

The overuse of antibiotics has inevitably led to an increase in antibiotic residues in environmental media, with evident regional variations. Asia ranks first in the world for antibiotic usage, with China being the largest producer and consumer of antibiotics [25]. Research indicates that the types and concentrations of antibiotic residues found in the aquatic environments of Asian countries exceed those detected in European and American nations [26]. Common antibiotics identified in China’s water bodies include *β*-lactams, QNs, TCs, and SAs [27]. Table 1 presents the classification and physicochemical properties of antibiotics. The concentrations of SAs, such as sulfamethoxazole (SMX), sulfadiazine (SDZ), and sulfamethazine (SMZ), typically range in the order of μg/L. Furthermore, comparative data reveal that the average concentration of antibiotics in water within the Asia–Pacific region is 17.7 mg/L, which is significantly higher than that recorded in Africa (11.3 mg/L), the United States (0.9 mg/L), and Europe (0.4 mg/L) [28]. More critically, the accumulation of antibiotics in soil, sediment, and sewage sludge is more pronounced than in aquatic environments [15], thereby exacerbating ecological and health risks.

### 2.2. Main Pollution Sources of Antibiotics

#### 2.2.1. Aquaculture

**Figure 1:**
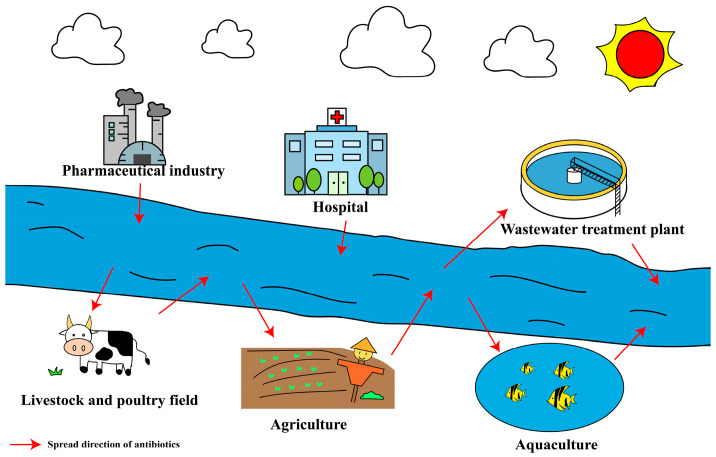
Sources and pathways of antibiotics in environmental media.

Antibiotic contamination in waters originates from a wide range of sources (Figure 1) [48]. Among these sources, aquaculture stands out as one of the fastest-growing sectors globally; however, it faces a significant challenge regarding the use of antibiotics [49]. FAO projects that aquaculture fish production may reach 109 million tons by 2030, with 89% of this output expected to come from Asia [50]. Due to the lower production efficiency and the higher cost of manpower and products in the traditional aquaculture modes, the intensive breeding approaches are commonly adopted to enhance economic benefit. However, the intensive aquaculture has also resulted in increased antibiotic usage. Data indicates that antibiotic concentrations in aquaculture systems typically range from μg/L to ng/L, and although there are some regional variations, the types of antibiotics present are generally consistent [15].

The use of antibiotics in aquaculture has both advantages and disadvantages. In the short term, it can enhance the productivity of aquatic animals while preventing disease outbreaks. However, a significant portion of antibiotics is excreted in the form of urine and feces due to their low absorption and utilization rates in these animals. More concerning is the potential for antibiotics to propagate through the food chain (e.g., antibiotics->fishes->human) or food web (e.g., antibiotics->fishes->human; antibiotics->plankton->fishes->human), accumulating in the human body and ultimately jeopardizing health. Limbu et al. [51] found that a moderate amount of antibiotics can promote the healthy growth of fish. However, excessive use or prolonged exposure can lead to the proliferation of ARGs and the emergence of antibiotic-resistant bacteria (ARB), impairing the physiological functions of animals [51,52]. Zhou et al. [53] demonstrated that the immune system of zebrafish was compromised when exposed to antibiotics, further confirming the antibiotic dose-dependent effect.

#### 2.2.2. Livestock and Poultry Field

The field of livestock and poultry breeding faces significant challenges of antibiotic risks. For instance, high-density pig and chicken farms have often drawn considerable attention due to the issue of antibiotic residues in feces and wastewater [54,55,56,57]. The concentrations of antibiotics in feces can range from 1 μg/kg to 100 mg/kg, while those in wastewater vary between 1 ng/L and 10 μg/L, with TCs being the most prevalent. In contrast, ML antibiotics exhibit the lowest concentrations [54]. These findings highlight a stark difference in the characteristics of antibiotic residues between livestock/poultry breeding and aquaculture systems. Despite the residual risk of antibiotics, it is important to note that some countries still permit the use of antibiotics as growth promoters in animal production [58,59,60].

### 2.3. Hazards of Antibiotic Overuse

As a new kind of contaminant exhibiting both biological toxicity and environmental persistence, antibiotics pose a significantly higher risk compared to conventional organic pollutants [61]. The detrimental effects are manifested in two ways: firstly, antibiotics can directly induce ecological toxicity; secondly, they facilitate the spread of ARGs. Only a small fraction of antibiotics is metabolized after entering the animal or human body, with approximately 50–90% being excreted into the environment as either unchanged drugs or metabolites [62].

#### 2.3.1. Generation and Spread of ARGs

Under antibiotic pressure, the cross-species transmission of ARGs through MGEs, such as plasmids and transposons, has emerged as a significant threat. Plasmids that carry ARGs in the water bodies of the Yangtze River Basin can be transferred across different bacterial genera via the T4SS, with aquaculture wastewater identified as a primary pollution source [5]. Jia et al. [63] discovered that antibiotic residues in the river system exhibited a significant co-occurrence with ARGs and ARB, further underscoring their potential hazards. Notably, global warming may accelerate the spread of ARGs and facilitate the proliferation of ARB through both direct and indirect pathways, highlighting the potential links among climatic factors and the drug resistance crisis [64].

#### 2.3.2. Microbial Community Imbalance

TCs that enter the soil through feces can significantly inhibit the function and abundance of key microbial communities, leading to a reduction in the nitrogen-fixing bacteria activities by 40–70% and a decrease in the nitrifying bacteria abundance by 50%, ultimately resulting in a 30% reduction in soil nitrogen cycling efficiency. Drug-resistant bacteria, such as methicillin-resistant *Staphylococcus aureus*, can survive in soil for two to three times longer than their non-resistant counterparts, thereby increasing the risk of transmission through the food chain via crop root systems [65]. Plasmids carrying the NDM-5 resistance gene, such as pX3_NDM-5 found in hospital wastewater, can be transferred across genera to 12 different phyla. This transmission disrupts microbial community structure and leads to a loss of diversity among native flora in aquatic environments [66].

#### 2.3.3. Destruction of Ecological Systems

Antibiotic residues can cause systemic damage to water, soil, and ecosystems. Studies have reported that the antibiotic levels in the water of the Yangtze River Basin range from 2.05 to 111 ng/L, while in the sediment, they are from 0.57 to 57.9 ng/L. Additionally, antibiotic residues of SAs and QNs are generally detected at the μg/L level [67]. Furthermore, regional pollution differences from antibiotics are particularly pronounced, with the antibiotic emission intensity in the Yangtze River Delta reaching as high as 60 kg/km^2^, significantly exceeding levels found in European and American countries. Moreover, it proved TC antibiotics could induce chronic toxic effects in tilapia, disrupt energy transfer within the food chain, and destabilize the structure of the aquatic food web [68].

## 3. Overview of ARGs Pollution in the Environment

There are two mechanisms for the generation of ARGs: one involves mutations within the bacterial genome, while the other occurs through the acquisition of external DNA [69,70]. Unless new antibiotics with novel structures or action mechanisms are developed, the existing drugs will continue to facilitate the spread of ARGs [71]. Since ARGs can be transmitted between environmental microbes and the human body through HGT, Pei et al. [72] first defined them as novel environmental pollutants in 2006. Unlike traditional pollutants, ARGs not only persist in the environment but can also be transferred to the genomes of environmental microbes or human symbiotic bacteria, thereby expanding the ecological risks. Current studies mainly focus on the types of high-frequency ARGs detected in soil and water environments, such as SAs ARGs (*sul*1, *sul*2, and *sul*3), TCs ARGs (*tet*A, *tet*B, and *tet*C), and MLs ARGs (*erm*A and *erm*C). Table 2 shows the action mechanisms of antibiotic resistance and related ARGs, which have been widely distributed across various environments, including rivers, lakes, oceans, sewage, soil, sludge, and air [24,73,74]. The ARGs can spread through both HGT and vertical gene transfer (VGT) diffusion. HGT occurs via conjugation (plasmid transfer), transduction (phage-mediated), and transformation (cell-free DNA uptake). ARGs are captured by MGEs and integrated into the genome, forming stable genetic resistance. In contrast, VGT relies on the inheritance of ARGs from parents to offspring.

Sub-inhibitory concentrations of antimicrobial agents can activate bacterial stress metabolic pathways and enhance the transfer efficiency of HGT. While reducing antibiotic concentrations can decrease the abundance of ARGs, studies have shown that ARGs can still persist in environments devoid of antibiotic exposure, which suggests that the MGEs carrying ARGs can sustain transmission independently of antibiotic stress [74]. Although antibiotic pressure drives bacteria to acquire exogenous ARGs through HGT and integrate them into their genomes, the autonomous transfer capabilities of MGEs enable ARGs to persist for extended periods without selective pressure. Therefore, it is difficult to completely halt the transmission of ARGs by merely controlling antibiotic use, and it is necessary to simultaneously intervene in the environmental migration pathways of MGEs.

### 3.1. Pollution Sources of ARGs

#### 3.1.1. Aquaculture

Aquaculture systems have emerged as significant reservoirs of ARGs and ARB due to the extensive use of antibiotics [92]. QNs, such as enrofloxacin (ENR), activate the bacterial SOS repair system at an ambient concentration of 0.1 μg/L, inducing the enrichment of ARGs in the gut of crayfish and in sediment, also accelerating their cross-species transmission. In China, *sul*1, *sul*2, *tet*A, and *tet*M were identified as the most prevalent ARGs in aquaculture environments, comprising 37% and 28% of the total ARGs, respectively, followed by *qnr*S, *bla*_TEM_, and *bla*_CTX-M_ [93]. ARGs are widespread in water, sediment, and organisms within aquaculture systems, primarily consisting of extrachromosomal MGEs, such as plasmids and integrons. Notably, the detection rate of the integron *intl*1 reached as high as 90%, significantly enhancing the HGT of ARGs [93]. *Intl*1 and transposons capture and recombine ARGs to form drug resistance gene cassettes, thereby facilitating the rapid dissemination of ARGs within microbial communities [93]. Furthermore, ARGs can be transferred to ARB, and it was reported that the abundance of the *tet*M gene in the gut microbiota of farmed fish was 2 to 3 orders of magnitude higher than that found in the surrounding water, which may subsequently enter the human body through the food chain [93].

#### 3.1.2. Wastewater

Wastewater discharged from wastewater treatment plants (WWTPs) carries a high abundance of ARGs and ARBs, which can directly contribute to the accumulation of ARGs in rivers [94]. A study reported the detection of 1030 ARG subtypes in the downstream river of a pharmaceutical factory in China, with the total abundance of *sul*1 and *sul*2 being significantly higher than that found in the WWTPs [95]. Metagenomic analysis further confirmed the carbapenem and *β*-lactamase genes present in WWTP were homologous to those found in hospital settings, which underscored the risks and potential harm associated with HGT [96]. Studies showed that ENR with sub-inhibitory concentration increased the abundance of ISCR1 elements by 4-fold in crayfish culture wastewater, which could carry the *bla*_CTX-M-15_ gene and significantly accelerate the HGT transmission of ARGs [95]. In addition, the abundance of phage-associated MGEs in effluent from WWTPs increased significantly compared to that in influent water (from 12.68% to 21.10%), indicating that the role of phages in the transmission of ARGs might have been overlooked for an extended period [97,98]. It is important to note that some environmental interventions may yield varying effects. While *Chlorella* extracellular polymers could reduce the bacterial toxicity of SMX, they also promoted integron-mediated transmission of ARGs, resulting in a substantial increase in *sul*1 abundance in water [98].

#### 3.1.3. Animal Husbandry

It is estimated that approximately 63,000 tons of antibiotics are used in global livestock production each year, and this number is expected to increase to 106,000 tons by 2030 [99,100]. *Tet*A, *tet*Q, *sul*1, and *sul*2 account for 60% to 85% of ARGs found in the feces of pigs and cattle; the abundance of ARGs can reach 10^6^ copies/g and further increase following manure application [95,100]. It is important to note that SA ARGs may increase rather than decrease during the composting process, aggravating the environmental risks [101]. Long-term manure application has been shown to significantly enhance both the diversity and abundance of soil ARGs; in a field subjected to 10 consecutive years of chicken manure application, the number of ARG subtypes rose to 615, with 44 genes enriched more than 1000 times and closely associated with mobile genetic elements such as IncP-1 type plasmid [95]. Concurrently, manure application resulted in a 40% reduction in the abundance of *Actinobacteria* in soil, while the abundance of *Proteobacteria* increased, which weakened the niche competition of microbial communities and further promoted the spread of ARGs [100]. Similarly, in soils with long-term pig manure application, the proportion of *Proteobacteria* increased from 18.7% to 25.8%, further aggravating the spread of ARGs [102].

#### 3.1.4. Soil

Soil serves as a crucial reservoir for ARGs in the environment, and the extent of soil pollution is closely linked to agricultural practices, industrial emissions, and wastewater irrigation. Pharmaceutical wastewater, as a source of industrial pollution, contributes to an increase in the proportion of *sul*1, *sul*2, and *qacE*Δ*1*. Additionally, microplastics significantly enhance the abundance of ARGs in soil through the adsorption of antibiotics and heavy metals (such as Cu^2+^), thereby promoting integron-mediated gene transfer [103]. The biofilm environment on the surface of microplastics is particularly conducive to the co-occurrence of ARGs and MGEs, accelerating the spread of ARGs across different media [103,104]. The input of soil organic carbon can regulate key genes in the pyruvate/acetyl-CoA metabolic pathway, such as *ack*A and *pta* genes, which facilitate the evolution of microbes from stress-tolerant to highly resistant and promote the enrichment of ARGs [101].

### 3.2. Transmission Mechanisms of ARGs

**Figure 2:**
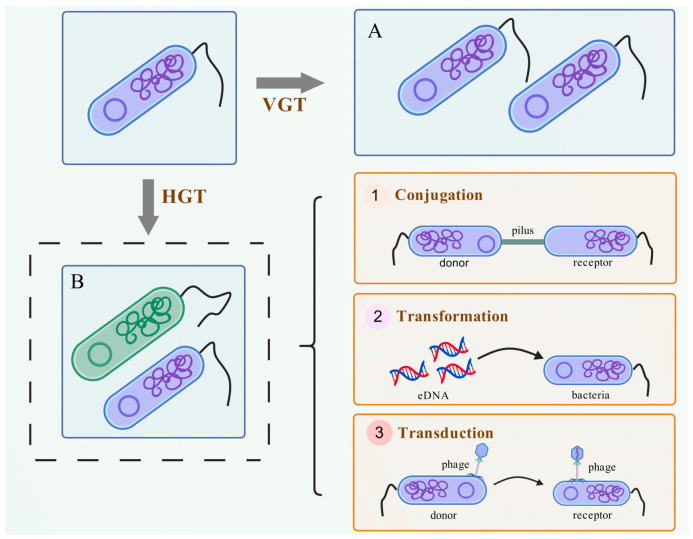
The transmission mechanism of ARGs in bacteria. (**A**) Bacteria transmit ARGs from parent to offspring via VGT. (**B**) Bacteria achieve the transfer of ARGs between different strains via HGT (e.g., conjugation, transformation, and transduction). The figure is created with BioGDP.com [106].

ARGs are primarily transmitted through HGT and VGT in microbes (Figure 2). HGT serves as the primary mechanism for the transmission of ARGs, which is predominantly facilitated by MGEs such as plasmids, transposons, integrons, and insertion sequences. This process includes plasmid-mediated conjugative transfer, extracellular DNA-mediated natural transformation, and phage-mediated transduction [105].

A few plasmids are linear, such as those found in *Borrelia*, *Streptomyces*, *Nocardia*, and *Rhodococcus*; however, most plasmids are circular and widely distributed among bacteria, and extrachromosomal DNA can autonomously replicate within host cells [107,108,109]. Additionally, plasmids serve as a “bridge” for gene transfer, facilitating gene exchange between different species through conjugative transfer. This process accelerates bacterial evolution and the diffusion of ARGs, which has a profound impact on ecosystems [110]. Integrons can be located on chromosomes or plasmids, and their core structure consists of the integrase gene (*int*l), an attachment site, and a promoter. The integrase encoded by the *int*l gene can insert gene cassettes carrying ARGs into the attachment site of the integron. This process enables the expression of resistance genes within the gene cassette through the promoter, allowing bacteria to acquire antibiotic resistance [110].

Transposons are DNA sequences that can move within or between genomes through type I and type II transposons. These elements often carry transposase genes and inverted repeats (IRs), which can facilitate HGT and genome rearrangement, driving bacterial evolution [111]. The simplest type II transposon, known as an insertion sequence (IS), is typically less than 2.5 kb in length and is restricted to its own movement, lacking additional functional genes. It can autonomously relocate, leading to gene inactivation, chromosomal rearrangement, or the activation of promoters, which can trigger a series of genetic effects [112].

Plasmids serve as vectors for cross-host transmission. Integrons are integrated into multiple gene cassettes, forming functional gene clusters. Transposons and IS facilitate gene recombination and enhance gene plasticity. These four types of MGEs work in concert to promote HGT and drive the evolution of bacterial resistance through their distinct characteristics and mechanisms.

#### 3.2.1. Conjugation

Conjugation is a gene transfer process mediated by MGEs, which requires direct contact between donor bacteria and recipient bacteria to form conjugation channels. Plasmids carry *tra* gene clusters that encode fimbrial proteins and DNA transferases, which regulate the conjugation process. For example, the IncHI2-type MDR plasmid transfers single-stranded DNA to recipient bacteria through the T4SS, facilitating the spread of ARGs [113]. T4SS, a multifunctional transmembrane channel found in Gram-positive bacteria, Gram-negative bacteria, and archaea, is capable of delivering large molecules, including DNA and proteins, into target cells [114]. The typical representative is the VirB/VirD4 system of *Agrobacterium tumefacien,* which is encoded by the Ti plasmid and facilitates the transfer of host genes [115].

Metagenomic analysis reveals the *sul*1 and translocation enzyme genes, such as *tnp*A, are associated with the localization phenomenon. This strongly confirms that MGEs mediate the widespread dissemination of ARGs within microbial communities [116]. Meanwhile, the carbapenemase gene (*bla*_OXA-48_), carried by IncF-type plasmids in hospital wastewater, is transferred to *Enterobacteriaceae* bacteria through conjugation, resulting in the formation of MDR bacteria, which exacerbates the challenges of preventing and controlling clinical infections [117].

#### 3.2.2. Transformation

Transformation is the process by which bacteria actively take up free DNA and integrate it into the genome, and its efficiency is jointly regulated by DNA stability, bacterial competence, and environmental conditions. Studies have found that only 3.36% of the plasmids in sewage sludge released phage cracking by the joint and the host DNA, mainly through the transmission route of ARGs [118]. Furthermore, in soil contaminated with carbendazim residues, the *sul*1 gene was taken up by *Pseudomonas* through transformation, and its abundance was positively correlated with the IS26 transposon, indicating that MGEs play a crucial role in this transformation process [119]. Extracellular polymers (EPS) in biofilms play an important role in the transformation process, as they can prolong the half-life of free DNA by adsorbing DNA and buffering nuclease activity. In soil exposed to TC, EPS secretion increased by 71.39%, resulting in a 2-fold increase in *tet* gene transformation efficiency. However, the addition of nano zero-valent iron (nZVI), which is a zero-valent iron particle with a particle size of 1–100 nm, has a high specific surface area, strong reducibility, and excellent adsorption performance and can decrease EPS secretion by 71.46% and suppress conversion efficiency [119]. In addition, ROS generated by photocatalysis damages the cell membrane to release cytoplasmic granules. During the photocatalytic treatment of water bodies, transformation accounts for more than 50% of HGT, among which *tet*A diffuses through DNA fragments encapsulated by bacteriophages [118].

DNA in the environment exists in two forms: intracellular DNA (iDNA) and extracellular DNA (eDNA), which are closely related [120]. The iDNA primarily originates from microbes with intact cellular structures; when these microbial cells rupture due to environmental factors or physiological activities, iDNA is released into the extracellular environment and subsequently converted into eDNA. eDNA can be taken up by other microbes and integrated into their genomes through recombination, thus becoming part of iDNA and influencing the genetic characteristics of these microbes [120]. Mao et al. [121] found that bacteria in river sediments can efficiently absorb resistance genes associated with kanamycin (KAN) under antibiotic stress. In natural environments, the enrichment of eDNA in river mouth biofilms promotes the frequency of HGT [122]. Following disinfection with chlorine or chloramine, the ROS oxidative stress pathway can be activated, leading to the uptake of eDNA and the utilization of foreign proteins as a source of amino acids for cellular repair [123].

#### 3.2.3. Transduction

Phage-mediated transduction is a key mechanism for HGT; it can be categorized into generalized transduction and specialized transduction. In generalized transduction, lytic phages (e.g., P1) play a significant role during the host lysis cycle, and phages randomly package fragments of host DNA, including ARGs, into their capsids. Phage P1 can transfer host DNA fragments of up to 100 kb in length and carry multidrug resistance gene cassettes, such as *bla*_CTX-M_ and *qnr*B [70]. Specific transduction is mediated by lysogenic phages such as the lambda phage; it may carry adjacent host gene fragments due to an abnormal excision process. This results in the formation of heterozygous DNA particles that contain both phage DNA and host DNA. Unlike general transduction, specific transduction transfers only the genes adjacent to the prophage and requires a helper phage to provide the necessary replication functions for complete packaging.

Environmental stress regulates transduction efficiency through multiple pathways, with chemical stress and the biofilm microenvironment playing particularly prominent roles. Sublethal concentrations of fluoroquinolone antibiotics can activate the host’s SOS response, initiate the original phage lytic cycle, and increase the number of phage-carrying ARGs by 4-fold [124]. Additionally, the antibacterial agent named triclosan enhances ROS production and increases membrane permeability, thereby promoting phage adsorption efficiency and significantly enhancing the environment for phage-mediated gene transfer [124]. Meanwhile, biofilms help stabilize phage particles through EPS and eDNA, which can double transduction efficiency [119], underscoring the critical role of biofilms in the dissemination of ARGs.

## 4. Removals for Antibiotics and Degradation ARGs in Environments

Antibiotics are increasingly detected in environments, including wastewater, surface water, and soil. The presence of residual antibiotics in the environment contributes to the global AMR crisis by exerting selective pressures that promote the spread of ARGs and the proliferation of ARBs. Therefore, the development of efficient and sustainable technologies to remove antibiotics is essential to mitigate selection pressure and reduce the risk of AMR spread.

At present, the strategies to remove the pollution of antibiotics and to degrade the pollution of ARGs in environments are mainly divided into three categories: The first is the adsorption method, which uses porous carbon materials (e.g., biochar, activated carbons (ACs), carbon nanotubes (CNTs), and graphene) to adsorb antibiotics and ARGs through a variety of adsorption mechanisms. The second is AOPs, which degrade antibiotics and ARGs by generating highly reactive free radicals (e.g., ·OH and SO_4_^−^·), including photolysis, electrochemical oxidation (EO), and Fenton/Fenton-like oxidation. In addition, it also includes biological methods, such as microbial degradation and constructed wetlands (CWs). The former is the degradation of antibiotics by specific bacteria (e.g., *Pseudomonas*, *Bacillus*), fungi (e.g., white rot bacteria), and algae. The latter is to remove antibiotics and ARGs through the synergistic effect of plant absorption, substrate adsorption (e.g., biochar and zeolite), and microbial degradation, in which microbial communities are the core driving force.

### 4.1. Adsorption Method

#### 4.1.1. Adsorbent Materials

- Biochar

Biochar is a porous carbon material produced through pyrolysis at temperatures ranging from 300 to 800 °C under anaerobic or anoxic conditions [125]. Its structural characteristics include a high specific surface area (SSA), well-developed pores, a large pore size, and a rich array of surface functional groups [126]. These features contribute to its excellent adsorption performance, making it widely utilized in environmental remediation and soil enhancement. Typically, the proportions of C, H, O, and ash in biochar range from 60% to 89%, 9% to 36%, 1% to 7%, and 0.2% to 40%, respectively [127]. The adsorption performance of biochar is influenced by factors such as pyrolysis temperature, heating rate, residence time, and the type of raw materials used.

The high SSA and surface functional groups of biochar contribute to its adsorption potential. Naghipour et al. [128] used biochar prepared from pinecones as the adsorbent and found the removal efficiency of cefixime (CFX) reached 92% under conditions of pH 6.3, a CFX concentration of 50 mg/L, and a reaction time of 90 min. However, the inherent characteristics of original biochar often limit its practical application in adsorption devices due to an underdeveloped pore structure and a limited number of active sites [129]. Therefore, it is essential to optimize physicochemical properties of biochar through physical, chemical, and biological modifications to enhance its selectivity and adsorption capacity for pollutants [130,131,132]. Table 3 shows the removal of antibiotics and ARGs by different original and modified biochars.

Huang et al. [145] demonstrated that ball-milled biochar could efficiently remove SMX and sulfapyridine (SPY), achieving removal rates of 83.3% and 89.6%, respectively. Soaking biochar in an acidic solution effectively removed surface ash and metal ions while breaking down the fatty chain structure, thereby improving SSA adsorption [164,165]. The adsorption efficiency of ciprofloxacin (CIP) by biochar modified with H_3_PO_4_ increased from 65% to 91%, and the adsorption capacity rose from 43.48 mg/g to 62.50 mg/g [166]. Li et al. [167] modified corn cobs with KOH, achieving an adsorption efficiency of 98.52% for SMX. Substances such as KMnO_4_ and H_2_O_2_ can be employed to oxidize impurities on the surface of biochar, thereby increasing the SSA and oxidative functional groups. Qin et al. [168] demonstrated that biochar modified with 30% H_2_O_2_ exhibited excellent adsorption performance for SMX and SDZ under conditions of pH 4 and 35 °C. Additionally, various metal elements, such as Fe, Zn, and Mg, can also be utilized for modification. For example, modifying biochar with Zn can increase its active sites [169]. Zhang et al. [170] found that iron-impregnated biochar significantly improved adsorption capacity for SMX when activated with H_2_O_2_ compared to the original biochar.

Biological modification, namely biochar-based immobilization technology (BIT), involves colonizing functional microbes within the pores of biochar. This approach effectively addresses critical challenges in traditional microbial remediation, such as the rapid loss of bacteria and poor resistance to environmental stressors [171]. As a result, BIT not only significantly increases the survival rate of microbes but also enhances the degradation rate of pollutants [171,172]. Biochar, characterized by its multi-level pore structure (pore diameter 0.1–50 μm), high SSA (greater than 200 m^2^/g), and various surface functional groups, serves not only as a microbial carbon source but also mitigates toxic effects through the formation of physical barriers, making it an ideal microbial carrier. At present, there are three methods of BIT [173], with pore size, surface charge, and hydrophobicity of biochar being the main factors affecting microbial colonization. Yang et al. [174] utilized straw biochar as a carrier, significantly enhancing the degradation capacity of SMX by *Pseudomonas stutzeri* and *Shewanella putrefaciens*. In a comparative study, Zhang et al. [154] found that the chlortetracycline (CTC) removal rate of *Bacillus subtilis* immobilized with lonicerae slag biochar was 15.31% higher than that of *B. subtilis* immobilized with corn straw biochar, further confirming that the selection of raw materials has a substantial impact on BIT performance.

Biochars adsorb not only antibiotics but also ARGs. Wang et al. [175] reported that maize straw biochar could effectively inhibit the spread of *tet*M, *tet*O, and *erm*B in pig manure compost. Huang et al. [176] found that pig manure biochar could reduce the total relative abundance of *tet*W and *tet*L in compost by 12–20%. Modified biochar demonstrated superior performance in adsorbing ARGs; the total adsorption rate of ARGs in wastewater using *β*-cyclodextrin-modified biochar (*β*-BC) reached 88% [177]. The magnetic biochar/quaternary phosphonium salt significantly reduced the absolute abundance of ARGs in livestock wastewater [151]. Specifically, the absolute abundance of ARGs in the wastewater from pig farms and chicken farms decreased by 3.153×10^10^ and 3.014×10^9^ copies/mL, respectively [178]. Lian et al. [73] found that the adsorption capacity of nano-scale biochar to eDNA (278–296 mg/g) was more than 100 times higher than that of bulk biochar, and the binding ability of *amp*C and *erm*B was 50–100 times higher than that of bulk biochar.

- Activated Carbons (ACs)

ACs play an important role in the adsorption of antibiotics and ARGs due to their high SSA (usually >1000 m^2^/g) and controllable pore structure (Table 3) [179]. According to the morphological characteristics, AC is primarily categorized into powdered activated carbon (PAC) and granular activated carbon (GAC). The adsorption performance of AC can be significantly enhanced through physical, chemical, and biological modification [180]. Moussavi et al. [181] modified AC with NH_4_Cl, achieving a 99% removal rate of amoxicillin (AMX) at a pH of 6.0. Mechanistic studies reveal that the modified AC exhibits stronger π-π interactions and a greater number of hydrogen bond binding sites compared to ordinary AC [182,183,184].

Antibiotics can be effectively removed by AC, and its adsorption capacity depends on pore structure, surface chemical properties, and the environmental conditions [185]. Ahmed et al. [186] showed that the removal rates of 28 antibiotics, encompassing six categories such as penicillins and MLs, in both surface water and deionized water were 99.6% and 99.9%, respectively, when using PAC at a dosage of 20 mg/L and a temperature of 25 °C. The results highlighted the universal applicability of PAC to complex water bodies. Choi et al. [187] found that GAC displayed significant selectivity in the removal of TC antibiotics. Because of its strong hydrophobicity, TC is more readily adsorbed by GAC, leading to more efficient removal. In contrast, the removal efficiency of oxytetracycline hydrochloride (OTC-HCl) was relatively low, indicating variability in the adsorption capacity of GAC for different types of antibiotics.

AC can effectively adsorb and reduce the concentration of ARGs in water [188]. The adsorption process is influenced by multiple factors, including the transmission of ARGs between the aqueous phase and biofilm through HGT [189]. When aged biofilm detaches, ARGs are released back into the aqueous phase, significantly diminishing the adsorption efficiency of AC [190]. This dynamic process imposes certain limitations on the effectiveness of AC in eliminating ARGs. Currently, the mechanisms by which AC adsorbs ARGs remain inadequately understood and require further investigation.

- Carbon Nanotubes (CNTs)

Since CNTs were first reported in 1991 [191], their unique mechanical strength, electrical conductivity, and chemical stability have rapidly attracted significant attention (Table 3) [192]. Based on the number of layers, CNTs can be divided into single-walled CNTs (SWCNTs), double-walled CNTs (DWCNTs), and multi-walled CNTs (MWCNTs). Due to their electrical, chemical, and structural properties, CNTs have demonstrated potential in the water pollution control field [192].

The adsorption properties of CNTs primarily depend on their SSA, pore structure, and surface functional groups. Zhang et al. [193] systematically investigated the adsorption properties of hydroxylated MWCNTs (MWCNTs-OH), carboxylized MWCNTs (MWCNTs-COOH), and graphitized MWCNTs (MWCNTs-G) for SMX. The MWCNTs-OH exhibited the best SMX adsorption capacity due to the highest SSA (228.0 m^2^/g). The order of adsorption capacity was positively correlated with the SSA (MWCNTs-OH > MWCNTs-G > MWCNTs-COOH). In addition, Carabinero et al. [194] compared the adsorption effects of CNTs and ACs on CIP and found that the adsorption capacity of CNTs was significantly higher than that of ACs due to their superior electron donor ability and enhanced surface accessibility. SWCNTs have the advantage of high SSA, which facilitates the adsorption of antibiotics. Ncibi et al. [160] demonstrated that, with an SSA of 577.0 m^2^/g and a pore volume of 0.4260 cm^3^/g, SWCNTs could adsorb 375 mg/g and 520 mg/g of OTC and CIP at an initial concentration of 50 mg/L and 25 °C, respectively. The maximum adsorption capacities of unmodified SWCNTs, DWCNTs, and MWCNTs on OTC and CIP were 724 mg/g and 554 mg/g, respectively. The same study revealed that the ethanol desorption rate of CIP in the MWCNTs system reached 52%, while the desorption rate of OTC was generally lower than 3.3%, indicating that there is a stronger chemical bond between OTC and the functional groups of CNTs [160].

- Graphene

Graphene has been successfully synthesized through mechanical exfoliation since 2004. Due to its exceptionally high SSA, outstanding electron mobility, and tunable surface chemical properties, graphene has emerged as a cutting-edge material in the field of environmental pollution control [195,196]. Its derivatives can be modified through functionalization to enhance adsorption performance (Table 3). Graphene oxide (GO) incorporates oxygen-containing functional groups via the Hummers method, which significantly increases hydrophilicity and the density of chemically active sites [197]. However, excessive oxidation can disrupt the π-π conjugated structure, thereby limiting its capacity to adsorb hydrophobic pollutants. Reduced graphene oxide (rGO) mitigates the presence of some oxygen-containing groups through thermal or chemical methods, restoring the π-π conjugated structure and balancing adsorption capacity with conductivity [198]. In addition, the composite integration of graphene with other materials enhances the adsorption of antibiotics. When rGO is combined with magnetite, the adsorption capacities for norfloxacin (NOR) and CIP reach 22.20 mg/g and 18.22 mg/g, respectively, at pH 6.2 and 25 °C, with a magnetic separation efficiency exceeding 98% [199]. Additionally, Mn_3_O_4_ was combined with GO and modified using alkali treatment, significantly increasing the active sites of Mn^3+^ and enhancing its propensity to form strong coordination bonds with SA antibiotics; its adsorption capacity was found to be 2.3 times greater than that of the original GO [200]. Graphene-based materials have demonstrated advantages in high adsorption capacity, efficient recovery, and targeted adsorption in the realm of antibiotic removal through surface functional group modification and structural design [201].

Among its various forms, GO can help control the spread of ARGs by inhibiting their replication. The mechanism of GO primarily involves the adsorption of ARGs and its interaction with eDNA. GO can penetrate the double helix structure of DNA, causing damage, and the intermediate products generated can further enhance the adsorption capacity of GO [202]. The binding energy of GO to plasmid DNA bases inhibits the replication of ARGs, thereby reducing their abundance. Zou et al. [203] demonstrated that GO could inhibit bacterial uptake of SMX and the transfer of related ARGs.

#### 4.1.2. Adsorption Mechanisms

**Figure 3:**
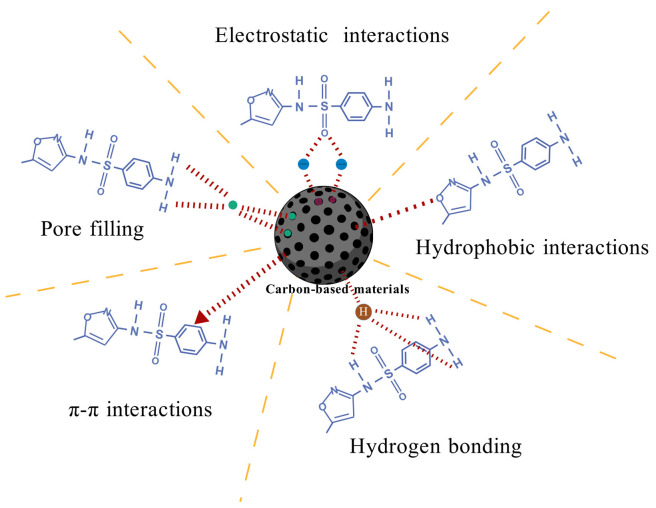
Adsorption mechanism of antibiotics on carbon-based materials (taking SMX as an example). The figure is created with BioGDP.com [106].

Carbon-based materials remove antibiotics and adsorb ARGs from the environment primarily through physical and chemical adsorption. Physical adsorption relies on electrostatic interactions, hydrophobic interactions, and pore filling (Figure 3) [149]. During the adsorption process of antibiotics, the pH of the solution can influence the charge on the surface of the antibiotic molecules. When the pH is lower than the pKa of the antibiotic and the isoelectric point of the adsorbent, the surface of the antibiotic becomes negatively charged, while the surface of the adsorbent is positively charged. This results in efficient adsorption through strong electrostatic interactions. In contrast to physical adsorption, chemical adsorption is more complex. Its defining characteristic is that antibiotics form chemical bonds, such as hydrogen bonds and π-π interactions, with the functional groups on the surface of the adsorbent [149], leading to the formation of stable surface complexes with high selectivity and stability.

- Electrostatic Interactions

Electrostatic interactions are one of the key mechanisms involved in the adsorption of antibiotics by biochar and other carbon-based adsorbents. The electrostatic attraction or repulsion between the surface charge of the adsorbents and the ionized state of the antibiotic molecules plays a crucial role in this process. Under neutral pH conditions, the surface of algal biochar carries a positive charge, which facilitates electrostatic interactions with the negatively charged ionization state of TC, significantly enhancing adsorption efficiency. However, in an alkaline environment, the adsorption efficiency decreases due to electrostatic repulsion and competition from OH^−^ for adsorption sites [204].

- Hydrophobic Interactions

Hydrophobic interaction refers to the weak force that drives the spontaneous aggregation of non-polar groups in an aqueous environment, resulting from the repulsion of water molecules. In the process of antibiotics adsorption by biochar, hydrophobic interactions often work synergistically with π-π interactions or electrostatic interactions. For instance, hydrophobic TC binds to the hydrophobic regions of straw biochar through its non-polar groups, significantly enhancing the adsorption efficiency of TC [205,206].

- Pore Filling

Pore filling refers to the process by which adsorbate molecules enter and occupy the pores of adsorbents. The pore size of adsorbents and the compatibility of adsorbate molecules significantly influence the adsorption rate of these materials [207]. Micropores and mesopores are particularly important as they serve as the primary sites for adsorption due to their high SSA. Additionally, the pyrolysis temperature plays a crucial role in determining pore size. As the pyrolysis temperature increases from 500 °C to 900 °C, the pore volume of sludge biochar rises from 0.056 to 0.099 cm^3^/g, resulting in enhanced adsorption capacity [208]. However, excessively high temperatures may cause the collapse of the pore structure in wheat straw biochar due to a decrease in the degree of graphitization, ultimately reducing its adsorption performance [209].

- Hydrogen Bonding

The hydrogen bonding interactions primarily involve -OH, -NH_2_, and other functional groups that form hydrogen bond structures with the surfaces of pollutants. The formation of hydrogen bond structures between the -NH_2_ of SAs and the -OH or -COOH on the surface of biochar can significantly influence adsorption performance [210,211]. Fourier transform infrared spectroscopy (FTIR) and X-ray photoelectron spectroscopy (XPS) showed that during the adsorption of OTC-HCl on the MWCNTs-CuNiFe_2_O_4_ composite, hydrogen bond interactions between C-O and antibiotic molecules on the surfaces of the adsorbents enhance their adsorption and binding [212,213]. Furthermore, hydrogen bonds also play a role in the adsorption process of DNA on carbonaceous materials [73,214].

- π-π Interactions

The π-π interactions are one of the fundamental mechanisms by which adsorbents capture aromatic antibiotics. This interaction primarily involves the π-electron interactions between the adsorbents and the aromatic rings of antibiotics. Antibiotics typically contain benzene rings, while the carbon skeleton structure of biochar is stable. The combination of these two components can significantly enhance the adsorption capacity. Zhou et al. [215] found that the -C=O on the surface of magnetic biochar modified by acid/base can function as π-electron acceptors, forming π-π interactions with the benzene ring in TC, which predominates the adsorption process. Similarly, Ninwiwek et al. [216] noted that SMX, due to its amino and oxygen-containing heterocyclic structure, acts as a π-electron acceptor, thereby enhancing its binding to adsorbents such as biocarbon through π-π interactions. Importantly, this mechanism is also significant for the adsorption of eDNA by biochar. Fang et al. [217] observed that after biochar adsorbed eDNA, the levels of C=C initially decreased and then increased, confirming the π-π interactions between biochar and the bases of eDNA, and found that this interaction was related to the graphitization degree of biochar [218].

### 4.2. Chemical Methods (AOPs)

AOPs are technologies that degrade organic pollutants by generating highly reactive free radicals, such as ·OH and SO_4_^−^·. AOPs are characterized by their strong oxidation potential, non-selectivity, and efficient mineralization, making them particularly suitable for the removal of refractory antibiotics [219,220]. The action mechanisms can be categorized into homogeneous and heterogeneous reactions, wherein antibiotics are decomposed into smaller molecular intermediates through the strong oxidative capacity of ROS [221]. Hydroxyl radicals have been utilized in wastewater treatment since 1980; the goal is to convert organic substances that are resistant to biological degradation into non-toxic inorganic compounds, such as CO_2_ and H_2_O [222,223]. With their universal adaptability and strong oxidative characteristics, AOPs have become a primary method for degrading antibiotics and ARGs. AOPs include photolysis, electrochemical oxidation, Fenton/Fenton-like oxidation, persulfate oxidation, ozone oxidation, etc. (Table 4) [224,225,226].

#### 4.2.1. Photolysis

Photolysis is a degradation technology that utilizes light energy to break chemical bonds or alter the molecular structure of antibiotics. This technology employs solar energy or artificial light sources to convert antibiotics into easily processed intermediates. Snowberger et al. [234] found that the direct photolysis of CIP at pH levels of 5, 7, and 9 significantly reduced its ecotoxicity, indicating that pH may alter the state of antibiotic molecules. Dai et al. [235] reported that the direct photolysis efficiency of TC was low; its degradation efficiency was significantly improved in the presence of a photosensitizer. QNs, TCs, and chloramphenicol with conjugated structures can act as both photo-absorbents and photo-sensitizing agents; these antibiotics generate ROS through energy transfer or electron transfer processes, subsequently initiating oxidative degradation reactions [236,237]. Environmental factors play an important role in regulating the photolysis reaction. Nitrate can generate ·OH during the photolysis, which significantly enhances its self-sensitization photolysis process, and the promoting effect is positively correlated with nitrate concentration [238].

The degradation of ARGs is mainly achieved through the dual photolysis mechanism in surface flow CWs. The direct photolysis mechanism involves the degradation of genetic material by disrupting the chromophores (such as DNA and proteins) of microbes when exposed to UVB radiation in the range of 280–320 nm [239]. In contrast, the mechanism of indirect photolysis is more complex. Dissolved organic matter (DOM) present in wastewater, along with microbial chromophores, acts as photosensitizing agents that generate ROS such as ^1^O_2_ and ·OH under illuminated conditions. These ROS effectively degrade ARG fragments through nonspecific oxidation [240].

#### 4.2.2. Electrochemical Oxidation (EO)

EO is an advanced oxidation technology that relies on electrochemical reactions. By applying external voltage and current in the electrolyzer, pollutants are driven to undergo oxidation–reduction reactions on the electrode surface, ultimately resulting in their mineralization into CO_2_ and H_2_O, etc. [241]. During electrochemical oxidation, pollutants are degraded through direct action and indirect action. Direct action means that the pollutant directly loses electrons on the anode surface to be oxidized, and the rate is controlled by the electrode material and the pollutant oxidation potential [242]. In indirect action, ions in the electrolyte (such as Cl^−^ or SO_4_^2−^) at the anode form secondary oxidants (such as Cl_2_ and H_2_O_2_) [243]. Taking metronidazole as an example, its degradation mainly depends on indirect oxidation mediated by ·OH, and the reaction rate is related to the production rate of ·OH [244].

Electrochemical oxidation inhibits the spread of ARGs through physical destruction and chemical degradation [245,246]. Wang et al. [247] used TiO_2_ nanotubes as anodes to effectively treat *Escherichia coli* strains carrying *tet*A and *sul*1, resulting in a significant reduction in ARGs abundance and inactivation of the host bacteria. Similarly, Wang et al. [248] employed Ti_4_O_7_ as an anode to treat MDR *Salmonella* Typhimurium strains carrying *te*tG, *flo*R, and *sul*1, achieving a remarkable reduction in ARGs abundance, with proportions reaching up to 99%.

#### 4.2.3. Fenton/Fenton-like Technology

The Fenton reaction was discovered and proposed by British chemist H.J.H. Fenton in 1894. Its core mechanism is based on the chain reaction between Fe^2+^ and H_2_O_2_, which produces ·OH. The reaction mechanism is as follows [232,233,234]:

Fe^2+^ + H_2_O_2_ → Fe^3+^ +·OH + OH^−^ (1)

·OH + H_2_O_2_ → HO_2_·+ H_2_O (2)

Fe^2+^ +·OH → Fe^3+^ + OH^−^ (3)

Fe^3+^ + HO_2_·→ Fe^2+^ + O_2_ + H^+^ (4)

Fenton technology is primarily employed to efficiently degrade antibiotics through three mechanisms: hydrogen atom extraction, electron transfer, and π-π electrophilic addition [249,250]. Elmolla et al. [251] demonstrated complete mineralization of AMX within 2 h at a pH of 3.0. When the pH exceeds 4.0, Fe^3+^ precipitates as Fe(OH)_3_, leading to a reduction in reaction efficiency, which severely limits its engineering applications. To overcome the aforementioned limitations, homogeneous photo-Fenton reaction, homogeneous electro-Fenton reaction, and heterogeneous Fenton-like reaction technologies have emerged, primarily by introducing energy sources such as light, electricity, and ultrasound, or by utilizing non-iron catalysts like Cu and Mn to optimize reaction conditions [252,253,254]. Moreover, modified graphite felt (MGF) enhances cathode activity in electrochemical applications compared to raw graphite. Huang et al. [255] utilized MGF as the cathode, achieving a 95.62% degradation rate for CIP within 30 min, highlighting its significant potential for engineering applications.

Under acidic conditions, Fenton technology catalyzes H_2_O_2_ to produce ·OH through Fe^2+^ catalysis, which directly disrupts the DNA structure of ARGs and inactivates microbes [256]. The efficiency of this process is influenced by reaction time and pH [257]. Reaction time needs to be adjusted according to the specific genotype [258]. For instance, it took 120 min to achieve a 97% degradation of the gene associated with clarithromycin (CLR), while the SMX gene required a longer duration due to its structural complexity [259]. The pH must be strictly maintained below 4; otherwise, iron complexes may form, reducing the efficiency of ARG degradation. However, this technology has notable limitations. On one hand, the intermediates generated are highly toxic. On the other hand, excessive concentrations of H_2_O_2_ can inhibit microbial activity and promote the horizontal transfer of ARGs [260]. In practical applications, it is necessary to continuously monitor the relevant parameters of Fenton technology to balance treatment efficiency with the ecological safety concerning ARGs.

### 4.3. Biological Methods

#### 4.3.1. Microbial Degradation

As a green and sustainable treatment technology, microbial degradation has garnered increasing attention in the field of antibiotic pollution control (Table 5). Activated sludge (AS) is an important source of bacterial isolation due to its rich microbial diversity. Using antibiotics as the sole carbon source, various strains capable of degrading antibiotics can be isolated. Vijayaraghavant al. isolated *B. subtilis* from AS and found that it could achieve complete degradation of SMX within 10 days [261]. *Acinetobacter* sp. and *Microbacterium* sp. also demonstrate similar degradation potential [262,263]. The addition of a carbon source can further enhance the degradation efficiency of SMX by *B. subtilis* and *Alcaligenes faecalis* [264,265]. Fungi also exhibit unique antibiotic degradation potential. White-rot fungi degrade fluoroquinolone antibiotics through multiple synergistic interactions involving laccase, peroxidase, and cytochrome P450 enzymes, with the cytochrome P450 enzyme playing a crucial role in the early stages of degradation [266]. In soils, Arbuscular mycorrhizal fungi promote the proliferation of soil microbes by increasing the activities of soil enzymes [267]. Their mycelial secretion, glomalin, further stimulates microbial diversity and richness by degrading OTC [268]. Microbes in AS can transform ML antibiotics such as azithromycin (AZM), ERY, and CLR into more than 30 small molecular products through the synergistic catalysis of intracellular and extracellular enzymes [269,270].

When algae are exposed to antibiotics, the removal of these substances can be achieved through biosorption, bioaccumulation, and biodegradation. Biosorption mainly degrades antibiotics by adsorbing them onto the surface of algae. For example, the adsorption rate of 5 μmmol/L metronidazole by *Chlorella* can reach 100%, and the residual biomass of *Chlorella* after lipid extraction still exhibits adsorption performance for cephalexin [287,288]. Bioaccumulation refers to the transmembrane transport of antibiotics by algae, which can be removed by passive transport into algal cells, as demonstrated by Song et al. [289]. Biodegradation occurs through the catalytic action of intracellular enzymes that convert antibiotics into small molecules with low or no toxicity; for example, *Chlorella vulgaris* and *Scenedesmus obliquus* mainly rely on the biodegradation mechanism to degrade SMZ and ENR [290].

However, the efficiency of antibiotic degradation by algae is influenced by both the specific antibiotics and the algal species involved. For instance, *Scenedesmus* spp. can achieve a degradation rate of 83–100% for AMX, while their efficiency for cefradine (CED) ranges from 7 to 23% [291]. Similarly, the degradation rates of CIP by *Chlamydomonas mexicana*, *Chlamydomonas pitschmannii*, and *Chlorella vulgaris* are alarmingly low, ranging from 0 to 13% [292]. This indicates that the compatibility between the antibiotic physicochemical properties and the algae metabolic capabilities is a crucial factor in determining degradation efficiency. It is essential to consider the potential risk that microbial metabolic processes may promote the spread of ARGs through HGT and VGT, a phenomenon that requires further detailed evaluation.

#### 4.3.2. Constructed Wetlands

CWs, which offer environmentally friendly, cost-effective, and low energy consumption, can effectively remove antibiotic pollutants through the synergistic interactions of plants, microbes, and substrates. Based on flow regime and direction, CWs can be categorized into surface flow CWs (SF-CWs), horizontal subsurface flow CWs (HSF-CWs), and vertical subsurface flow CWs (VSF-CWs). HSF-CWs operate under hypoxic conditions and primarily depend on substrate adsorption and anaerobic degradation. The removal rates for TCs, fluoroquinolones, and MLs exceed 80%, while the removal rates for SAs and lincomycin (LIN) under aerobic conditions range from 40% to 60% [293]. VSF-CWs form an unsaturated substrate layer through intermittent water intake, combined with oxygen supplied by plant roots, resulting in an aerobic-anaerobic microenvironment. The removal rates for ceftiofur (CEF), trimethoprim (TMP), and clindamycin (CLI) range from 90% to 100% [294,295], underscoring their effectiveness in removing compound antibiotics.

In response to the pressing issue of antibiotic pollution, CWs can mitigate antibiotics through substrate adsorption, plant absorption and precipitation, and microbial degradation (Table 5). The removal efficiency of antibiotics can exceed 90% [296,297]. Among these mechanisms, microbes serve as the core driving force behind degradation; the stability of microbial community structure directly influences the effectiveness of antibiotic removal. Take SA antibiotics as an example; these antibiotic residues will inhibit the activities of desulfurizing and denitrifying bacteria, thereby interfering with the sulfur-nitrogen cycle process [298]. However, the phyla *Firmicutes, Proteobacteria*, and *Actinomycetes* found in CWs can effectively degrade them through metabolic regulation, highlighting the importance of microbial diversity in the removal of antibiotic contamination [299]. In addition, *Proteobacteria*, *Acidobacteria,* and *Bacteroidetes* have emerged as the dominant bacterial groups due to their possession of antibiotic degradation genes [300,301]. It is worth noting that *β-proteobacteria* within the *Proteobacteria* not only exhibit efficient antibiotic decomposition capabilities but also can inhibit the spread of ARGs through metabolic regulation. This offers new insights into addressing the global challenge of antibiotic resistance [302,303].

CWs have significant potential for reducing ARGs abundance, and their effectiveness is related to the type of CW and the characteristics of the substrate (Table 5). Studies have demonstrated that the reduction rate of the abundance of ARGs in SFCWs is generally low, typically ranging from 14.5% to 100% [304]. In contrast, HFCWs achieve a reduction rate exceeding 50% for the abundance of *sul*1 and *sul*2 through the synergistic effect of physical filtration and biological processes [305]. The reduction efficiencies in abundance surpass those of traditional WWTPs [306]. Results indicate that the reduction efficiency in abundance of ML ARGs by CWs is only 43%, which may be attributed to the residual levels of antibiotics [307]. In addition, the reduction rate in abundance of *sul*1 using a zeolite matrix is 85.6%, while the reduction rate in abundance of *sul*2 using a gravel matrix can reach 97% [308,309]. The reduction efficiency in the abundance of ARGs is closely related to plant-substrate synergy. The combination of large plants and porous substrates can create an effective system for reducing the abundance of ARGs [284,308]. It is important to note that under antibiotic stress, the abundance of ARGs significantly increases and becomes enriched in the biofilm associated with plant roots and leaves. Additionally, the exogenous introduction of heavy metals can promote the HGT of ARGs. While aeration can slightly enhance ARGs reduction in abundance, it may also lead to an increase in the abundance of certain ML ARGs [310].

## 5. Summary and Perspectives

The overuse of antibiotics has led to increased residual concentrations and induced ARG occurrences in environmental media, exacerbating the HGT of ARGs mediated by MGEs. Antibiotic contamination and the proliferation of ARGs have emerged as significant global environmental and public health challenges, posing potential threats to ecosystems and human health. The adsorption of antibiotics and ARGs from the environment can be effectively achieved through the adsorption of biochar, AC, CNT, and graphene; AOPs, including photolysis, EO, and the Fenton/Fenton-like technology; and biological methods, such as microbial degradation and CWs. Among current remediation technologies, carbon-based materials demonstrate effective adsorption of antibiotics and ARGs due to their unique physicochemical properties. AOPs generate highly reactive radicals to oxidize antibiotics and ARGs. Biological methods utilize microbial metabolic activities for antibiotic degradation, though their efficiency is significantly influenced by environmental factors and may paradoxically promote ARGs dissemination. CWs exhibit superior performance in mitigating antibiotic residues and reducing the risks of ARGs transmission by integrating substrate adsorption, plant uptake, and microbial degradation processes.

While existing antibiotic degradation technologies show promising removal efficiencies for both antibiotics and ARGs, several limitations persist in practical applications. Adsorption methods face challenges in adsorbent regeneration and secondary pollution. Although AOPs achieve efficient antibiotic mineralization, they suffer from high operational costs, toxic byproduct generation, and catalyst recovery difficulties. Microbial degradation efficiency remains constrained by environmental conditions and community structure. Moreover, current research predominantly focuses on single antibiotic removal rather than addressing complex antibiotic mixtures, failing to reflect the real environmental conditions. Future developments should prioritize composite antibiotic pollution scenarios, aiming to create more efficient, economical, and eco-friendly degradation technologies while optimizing process parameters to reduce application costs and environmental risks.

The environmental transmission of ARGs involves microbial interactions, HGT, and plasmid/integron-mediated cross-media transfer. Plasmids, transposons, and integrons can facilitate the widespread dissemination of ARGs among microbial communities through the conjugation, transformation, and transduction pathways. Environmental co-contaminants, such as sub-inhibitory antibiotics, microplastics, and heavy metals, further accelerate the propagation of ARGs by inducing ROS and enhancing cell membrane permeability. Although combined technologies can remove ARGs, achieving complete elimination of transmission risks remains challenging in natural environments. Breakthroughs in molecular mechanisms are imperative: multidisciplinary approaches employing metagenomics, transcriptomics, and metabolomics should be employed to investigate ARGs migration patterns and the regulatory mechanisms of T4SS key genes. Concurrent development of targeted intervention technologies is crucial, including MOF-based gene carrier capture materials, CRISPR-Cas9 gene editing for precise ARGs module modification, and phage therapy for the selective elimination of resistant bacteria to curb ARGs generation and dissemination.

It should be pointed out that the scope and frequency of environmental surveillance of antibiotics and ARGs are still limited, especially in some developing countries and remote areas, where systematic surveillance data are lacking to accurately assess the status and trends of antibiotic contamination. The public has insufficient awareness of the environmental pollution caused by the abuse of antibiotics and generally lacks both awareness and the self-restraint ability for the rational use of antibiotics, which are the important reasons for the difficulty in solving the antibiotic pollution problem. In the future, the construction of a global surveillance network and the level of surveillance technology should be strengthened to achieve real-time and dynamic monitoring of antibiotics and ARGs in the environment. It is necessary to strengthen the publicity and education of the public, disseminate relevant knowledge through multiple channels and forms, improve the public’s awareness of antibiotic pollution and ARGs, and promote the formation of a good atmosphere for rational use of antibiotics in the whole society. In addition, there still exist many shortcomings, such as antibiotic degradation efficiency not being high, ARG control being difficult, and the monitoring system being imperfect; therefore, it is essential to enhance interdisciplinary studies, develop more effective governance technologies and strategies, and strengthen the formulation and implementation of policies and regulations in the future to achieve effective control of antibiotic pollution and the spread of ARGs, protecting the ecological environment and promoting sustainable development.

## Figures and Tables

**Table 1:** The classification and physicochemical properties of antibiotics.

Types	Compound	CAS	Molecular Formula	Molecular Weight	pKa Values	Log Kow	References
Sulfonamides (SAs)	Sulfamethoxazole (SMX)	723-46-6	C_10_H_11_N_3_O_3_S	253.3	1.6; 5.7	0.89	[29,30]
	Sulfamerazine (SMZ)	127-79-7	C_11_H_12_N_4_O_2_S	264.3	2.24; 6.92	1.41	[31]
	Sulfadiazine (SDZ)	68-35-9	C_10_H_10_N_4_O_2_S	250.3	6.36	−0.0314	[32]
Tetracyclines (TCs)	Oxytetracycline (OTC)	79-57-2	C_22_H_24_N_2_O_9_	460.4	3.53; 7.25; 9.58	−0.9	[33]
	Chlortetracycline (CTC)	57-62-5	C_22_H_23_CIN_2_O_8_	478.9	7.435	−0.62	[34]
	Tetracycline (TC)	60-54-8	C_22_H_24_N_2_O_8_	444.4	3.3; 7.68; 9.69	−1.3	[35]
Macrolides (MLs)	Erythromycin (ERY)	114-07-8	C_37_H_67_NO_13_	733.9	3.06	8.9	[36,37]
	Azithromycin (AZM)	83905-01-5	C_38_H_72_N_2_O_12_	749.0	8.74	4.02	[36]
	Clarithromycin (CLR)	81103-11-9	C_38_H_69_NO_13_	748.0	8.99	3.16	[38]
*β*-lactams	Penicillin G (PEN)	61-33-6	C_16_H_18_N_2_O_4_S	334.4	2.74	1.83	[39]
	Amoxicillin (AMX)	26787-78-0	C_16_H_19_N_3_O_5_S	365.4	3.37; 8.96	0.87	[40,41]
Aminoglycosides (AGs)	Kanamycin (KAN)	59-01-8	C_18_H_36_N_4_O_11_	484.5	7.2	<−3	[42,43]
	Streptomycin (SM)	57-92-1	C_21_H_39_N_7_O_12_	581.6	--	−7.53	[43]
	Tobramycin (TO)	32986-56-4	C_18_H_37_N_5_O_9_	467.5	6.98	--	[44]
Quinolones (QNs)	Ciprofloxacin (CIP)	85721-33-1	C_17_H_18_FN_3_O_3_	331.3	6.09; 8.74	0.28	[45]
	Enrofloxacin (ENR)	93106-60-6	C_19_H_22_FN_3_O_3_	359.4	2.7−3.9	--	[46]
	Ofloxacin (OFX)	82419-36-1	C_18_H_20_FN_3_O_4_	361.4	5.97; 9.28	−0.39	[45]
	Levofloxacin (LEV)	100986-85-4	C_18_H_20_FN_3_O_4_	361.4	6.02; 8.05	−0.39	[47]

--: None. The inclusion criteria for the listed antibiotic types are based on their chemical structure.

**Table 2:** The action mechanisms of antibiotic resistance and related ARGs.

Types	Mechanisms	ARGs	References
Sulfonamides (SAs)	Similar in structure to para-aminobenzoic acid (PABA), competitively binding to dihydropteroic acid (DHP), preventing the synthesis of tetrahydrofolate (the conversion of DHP), and hindering the synthesis of nucleic acids	*sul*1, *sul*2, *sul*3, and *sul*4	[75,76]
Tetracyclines (TCs)	Specifically binding to the 30S subunit of bacterial ribosomes, preventing the binding of aminoacyl tRNA to mRNA complexes, blocking peptide chain extension, and interfering with protein synthesis required for bacterial growth	*tet*A, *tet*B, *tet*C, *tet*D, *tet*E, *tet*G, *tet*K, *tet*M, and *tet*W	[77,78,79,80]
Macrolides (MLs)	Binding to the peptide donor site (P site) of the 50S subunit of bacterial ribosomes, inhibiting the translocation or transfer of peptidyl tRNA, hindering peptide chain extension, and interfering with protein synthesis	*erm*A, *erm*B, *erm*C, *erm*D, *erm*F, *erm*G, *erm*T, *erm*Y, *mef*A, and *mef*E	[81,82,83]
*β*-lactams	Binding to penicillin-binding proteins (PBPs), inhibiting the transpeptidases in peptidoglycan synthesis, leading to structural defects in the cell wall, and causing bacterial expansion and lysis	*bla*_TEM_, *bla*_NDM_, *bla*_OXA_, *bla*_CTX-M_, and *bla*_SHV_	[84,85,86,87]
Aminoglycosides (AGs)	Combining bacterial ribosome 30S and the A-site on 16S to further interfere with protein synthesis	*aac*(3)-I, *aac*(6′)-I, and *aph*(3′)-I	[88,89]
Quinolones (QNs)	Inhibiting DNA helicase and topoisomerase IV, disrupting bacterial DNA replication, and interfering with normal DNA metabolism	*qnr*A, *qnr*B, *qnr*C, *qnr*D, *qnr*E, *qnr*S, and *qnr*VC	[90,91]

**Table 3:** The adsorption effects of different adsorbents on antibiotics and ARGs.

Adsorbents		Raw Materials	Antibiotics/ ARGs	Initial Concentration	Condition	Efficiency/ Uptake	References
Biochar		Calamus	ERY	50 mg/L	pH = 8.5; t = 1 h; T = 25 °C	325 mg/g	[133]
		Pomelo peel	TC	10 mg/L	pH = 8.5; t = 48 h; T = 25 °C	476.19 mg/g	[134]
		Rice husk	CIP	25 mg/L	pH = 6.0; t = 12 h; T = 25 °C	50.32 mg/g	[135]
		Pomelo peel	CTC	10 mg/L	pH = 8.5; t = 48 h; T = 25 °C	555.56 mg/g	[134]
		Sugarcane bagasse	TC	20 mg/L	pH = 8.5; t = 2 h; T = 25 °C	85.5 mg/g	[136]
		Cassava waste	OTC	1 mg/L	pH = 7.0; t = 24 h; T = 25 °C	2.43 mg/g	[137]
		Coffee grounds	SMX	0.5 mg/L	pH = 6.8; t = 24 h; T = 25 °C	0.13 mg/g	[138]
		Pinecone biochar	*sul*1, *tet*W	10 mg/L	t = 30 d; T = 20 °C	13–21%	[139]
		Sewage-sludge biochar	eDNA	100 mg/L	pH < 5.0; t = 5 h; T = 25 °C	1 mg/g	[140]
Physical modification	Steam activation	Burcucumber plants; H_2_O	SMZ	50 mg/L	pH = 3.0; t = 72 h; T = 25 °C	37.7 mg/g	[141]
		Bamboo; H_2_O	TC	100 mmol/L	pH = 5.0; t = 50 h; T = 25 °C	95.75%	[132]
	Heat treatment	Pinewood sawdust; 800 °C	TC	48 mg/L	pH = 5.0; t = 48 h; T = 25 °C	18.8-fold	[142]
		Softwood sawdust; 700 °C	TC	25 mg/L	pH = 6.8; t = 48 h; T = 25 °C	5.5–9.2-fold	[143]
	Ball milling	Poplar woodchips; 300 °C	ENR	20 mg/L	pH = 6.8; t = 3 h; T = 25 °C	93.4 mg/g	[144]
		Hickory chips; 450 °C	SMX	10 mg/L	pH = 6.0; t = 12 h; T = 25 °C; r = 250 r	83.3%	[145]
Chemical modification	Acid modification	Camellia oleifera shells; H_3_PO_4_	TC	25 mg/L	pH = 6.0; t = 4 h; T = 25 °C	451.5 mg/g	[146]
		Swine manure; HCl	SMZ	9 mg/L	pH = 3.0–9.0; t = 12 h; T = 25 °C	1.58 mg/g	[147]
	Alkali modification	Pomelo peel derived biochar; KOH	TC	40 mg/L	pH = 7.0 ± 0.5; t = 48 h; T = 25 °C	402.86 mg/g	[134]
		Pomelo peel derived biochar; KOH	CTC	40 mg/L	pH = 7.0 ± 0.5; t = 48 h; T = 25 °C	456.68 mg/g	[134]
	Oxidative modification	Rape stalk; H_2_O_2_	TC	20 mg/L	pH = 9.0; t = 22 h; T = 25 °C	42.45 mg/g	[148]
	Metal oxide and metal salt modification	Poplar wood chips; Fe_2_O_3_	NOR	50 mg/L	pH = 6.0; t = 24 h; T = 25 °C	38.77 mg/g	[149]
		Municipal wastewater sludge; ZnCl_2_	SMX	100 mg/L	pH = 3; t = 24 h; T = 25 °C	50.6 mg/g	[150]
		Magnetic biochar; quaternary phosphonium salt	Calf thymus DNA	100 μg/mL	pH = 7.0; t = 24 h; T = 25 °C	>92.7%	[151]
Biological modification		Reed charcoal and wheat bran; *Achromobacter and Parapedobacter*	TC	20 mg/L	W:V = 1:10; t = 24 h; T = 30 °C; r = 180 r	1.37-fold and 11.44-fold	[152]
		Straw magnetic biochar; *Mycolicibacterium* sp.	OTC	25 mg/L	W:V = 1:200; t = 24 h; T = 30 °C; r = 160 r	71.8%	[153]
		Honeysuckle residue-derived biochar; *Bacillus subtilis*	CTC	50 mg/L	pH = 7.0; t = 72 h; T = 30 °C; r = 180 r	78.35%	[154]
		Forsythia, erding and chrysanthemum; *Bacillus cereus*	CTC	50 mg/L	W:V = 1:10; t = 48 h; T = 30 °C; r = 180 r	82.34%	[155]
Activated carbons		Powdered activated carbon	CIP	2 mg/L	pH = 3.9; t = 48 h; T = 25 °C	291.96 mg/g	[156]
		Macadamia nut shells; NaOH	TC	600 mg/L	pH = 3.0; t = 3 h; T = 25 °C	455.33 mg/g	[157]
		Guava seeds; NaOH	AMX	800 mg/L	pH = 4.0; t = 4 h; T = 25 °C	570.48 mg/g	[158]
Carbon nanotubes		Multi-walled carbon nanotubes	CIP	20 mg/L	pH = 7.0; t = 0.5 h; T = 40 °C	73%	[159]
		Single-walled carbon nanotubes	OTC	50 mg/L	pH = 6.7–7.0; t = 26 h; T = 25 °C	375 mg/g	[160]
			CIP	50 mg/L	pH = 6.7–7.0; t = 26 h; T = 25 °C	520 mg/g	[160]
Graphene		Graphene-oxide	CIP	20 mg/L	pH = 5.0; t = 26 h; T = 25 °C	379 mg/g	[161]
			SMX	40 mg/L	pH = 5.0; t = 26 h; T = 25 °C	240 mg/g	[161]
			*sul*2	1.50 × 10^5^ copies/g	t = 18 d; T = 55 °C	76.12%	[162]
		Reduced graphene oxide	SMX	5 mg/L	pH = 6.0; t = 3 h; T = 25 °C	92%	[163]

OTC: Oxytetracycline; SMX: Sulfamethoxazole; CTC: Chlortetracycline; TC: Tetracycline; ERY: Erythromycin; AMX: Amoxicillin; CIP: Ciprofloxacin; ENR: Enrofloxacin; SMZ: Sulfamerazine; NOR: Norfloxacin.

**Table 4:** The degradation effects of AOPs on antibiotics and ARGs.

Types			Antibiotics/ ARGs	Initial Concentration	Condition	Degradation Efficiency	References
Photolysis			CAP	3.0 μmol/L	t = 0.5 h; T = 26 °C; pH = 5.7; dark light	20%	[227]
			Florfenicol	20 μmol/L	t = 80 h; T = 25 °C; pH = 7.0; solar irradiation	24%	[228]
Electrochemical oxidation		PbO_2_/Ti/Na_2_SO_4_	LEV	500 mg/L	Current density = 50 mA·cm^−2^; Voltage = 9.8 V; t = 2.67 h; pH = 7	98.41%	[228]
		Stainless steel/stainless steel/Peroxydisulfate	OFX	5 mg/L	Current density = 25 mA·cm^−2^; Voltage = 2.6–3.1 V; t = 1.5 h; pH = 4	89.6%	[228]
Fenton/Fenton-like technology	Fenton technology	Fe^2+^/H_2_O_2_	*int*I1, *sul*1, *tet*X	--	--	2.58–3.79 logs	[229]
	Photo-Fenton oxidation	UV/H_2_O_2_/Fe^2+^	CPFX	15 mg/L	Fe^2+^ = 0.05 mmol/L; H_2_O_2_ = 5.0 mmol/L; pH = 4.0	71%	[230]
		Fe^2+^/H_2_O_2_	*int*I1	0.5	Fe^2+^ = 0.1 mmol/L; H_2_O_2_ = 1.47 mmol/L; pH = 2.8; natural sunlight	23%	[231]
	Electro-Fenton oxidation	H_2_O_2_/Fe^2+^/Current	SAs	0.5 mmol/L	Fe^2+^ = 0.5 mmol/L; H_2_O_2_ = 7 mg/L; pH = 3.0	92%	[232]
	Heterogeneous photo/Electro-Fenton oxidation	UV/H_2_O_2_/Fe_3_S_4_	SMX	5 mg/L	Fe_3_S_4_ = 15 mg; H_2_O_2_ = 9.79 mmol/L; pH = 5.0	93%	[233]

OFX: Ofloxacin; LEV: Levofloxacin; SAs: Sulfanilamides; CAP: Chloramphenicol; CPFX: Ciprofloxacin Hydrochloride; --: None.

**Table 5:** The degradation effects of different biological methods on antibiotics and ARGs.

Types		Strain/ Plant	Antibiotics/ ARGs	Initial Concentration	Operating Condition	Degradation Efficiency	References
Bacterium		*Arthrobacter* *nicotianae* OTC-16	OTC	100 mg/L	t = 8 d; T = 30 °C; r = 180 r	98.5%	[271]
		*Klebsiella* sp. strain TR5	TC	200 mg/L	pH = 7.0; t = 36 h; T = 25 °C; r = 180 r	90%	[272]
		*Sphingobacterium changzhouense* TC931	TC	10 mg/L	pH = 7.0; t = 36 h; T = 30 °C; r = 150 r	87.38%	[273]
Fungi		*Trichosporon mycotoxinivorans* XPY-10	TC	800 mg/L	t = 7 d; T = 30 °C; r = 120 r	78.28%	[274]
		*Trametes villosa* and *Pycnoporus sanguineus*	CIP	2.5 mg/L	t = 24 h; T = 40 °C; r = 120 r; in the dark	25%	[275]
		*Pycnoporus* sp. SYBC-L10	OTC	500 mg/L	t = 5 min; T = 30 °C; r = 200 r	100%	[276]
		*Pleurotus ostreatus*	SMX	50 mg/L	t = 15 d; T = 25 °C; r = 120 r	74%	[277]
		*Phanerochaete chrysosporium*	SMX	10 mg/L	pH = 4.5; t = 10 d; T = 35 °C; r = 160 r	74%	[278]
Algaes		*Chlamydomonas* sp. Tai-03	CIP	10 mg/L	pH = 6.2; t = 9 d; r = 300 r; 2%CO_2_	65.05%	[279]
			SDZ	10 mg/L	pH = 6.2; t = 9 d; r = 300 r; 2%CO_2_	17.05%	[279]
		*Chlorella pyrenoidosa*	CED	50 mg/L	t = 24 h; T = 25 ± 1 °C; light/dark cycle = 12 h:12 h; 70 μmol∙photons∙m^−2^∙s^−1^	41.47 ± 0.62%	[280]
		*Spyrogira* sp.	STZ	200 μg/L	t = 20 d; T = 20 °C; light/dark cycle = 12 h:12 h; 15 μmol∙photons∙m^−2^∙s^−1^	36%	[281]
Constructed wetlands	SF-CWs	Phragmites australis	OTC	30 μg/L	t = 15 d	99 ± 0.27%	[282]
			CIP	30 μg/L	t = 15 d	97 ± 0.26%	[282]
			*sul*1, *tet*A, *tet*C, *tet*E, and *qnr*S	5.68 × 10^7^ copies/g	t = 30 d	77.8%	[283]
	HSF-CWs	Thalia dealbata Fraser	*sul*1	(1.26 ± 0.01) × 10^5^ copies/g	t = 7 d	70.0 ± 6.82%	[284]
			*sul*2	(9.17 ± 0.42) × 10^7^ copies/g	t = 7 d	47.2 ± 13.8%	[284]
		Phragmites australis	SAs	400 μg/kg	t = 3 d	95%	[285]
		Thalia dealbata Fraser	*flo*R	(2.16 ± 0.01) × 10^6^ copies/g	t = 7 d	88.2 ± 1.54%	[284]
			*cml*A	(1.30 ± 0.01) × 10^6^ copies/g	t = 7 d	80.9 ± 2.78%	[284]
	VSF-CWs	Pontederia cordata	TC	254.53 mg/L	t = 4 d	91%	[286]
			OTC	228.53 mg/L	t = 4 d	90%	[286]
		Thalia dealbata Fraser	*tet*O	(1.20 ± 0.02) × 10^3^ copies/g	t = 7 d	76.9 ± 8.56%	[284]
			*erm*B	(7.41 ± 0.05) × 10^4^ copies/g	t = 7 d	85.2 ± 3.30%	[284]

OTC: Oxytetracycline; SDZ: Sulfadiazine; TC: Tetracycline; CIP: Ciprofloxacin; CED: Cefradine; SAs: Sulfonamides; STZ: Sulfathiazole.

## Data Availability

No new data were created or analyzed in this study.
